# Editorial: Highlights in Cardiovascular Imaging: 2021

**DOI:** 10.3389/fcvm.2022.934668

**Published:** 2022-06-08

**Authors:** Djawid Hashemi, Sebastian Kelle, Christos V. Bourantas, Karthik H. Chandrasekharan

**Affiliations:** ^1^Department of Internal Medicine and Cardiology, German Heart Center Berlin, Berlin, Germany; ^2^Department of Internal Medicine and Cardiology, Charité – Universitätsmedizin Berlin, Corporate Member of Freie Universität Berlin and Humboldt Universität zu Berlin, Berlin, Germany; ^3^Deutsches Zentrum Für Herz-Kreislaufforschung (German Centre for Cardiovascular Research), Berlin, Germany; ^4^Department of Cardiology, Barts Heart Centre, Barts Health National Health Service (NHS) Trust, London, United Kingdom; ^5^Centre for Cardiovascular Medicine and Devices, William Harvey Research Institute, Queen Mary University London, London, United Kingdom; ^6^Institute of Cardiovascular Sciences, University College London, London, United Kingdom

**Keywords:** cardiovascular, artificial intelligence (AI), imaging, atherosclerosis, heart failure

## Introduction

This Research Topic presents the highlights in Cardiovascular Imaging and includes a range of studies and reviews across multiple imaging modalities and subspecialities, that describe recent advances in established modalities, including echocardiography, cardiac computed tomography (CT), and cardiac magnetic resonance imaging (CMR). It also presents novel applications of micro-CT and X-ray phase-contrast imaging, as well as innovative techniques e.g., 3D holograms, and discusses the use of artificial intelligence (AI) in cardiovascular imaging. We selected the studies from the most read and downloaded articles in 2021 in this journal. The selection of articles by the authors aimed to highlight novel techniques and applications across the broad range of cardiovascular imaging.

## Artificial Intelligence

Artificial intelligence (AI) algorithms have revolutionized image analysis and improved the predictive value of imaging parameters as shown by Bard et al. as well as Kameshina et al.

Bard et al. successfully compared an AI approach to both segment and quantify pericardial adipose tissue (PAT) by CMR in a dataset from the UK Biobank. The results were compared to the gold standard of PAT quantification by cardiac CT. This study showed that the CMR-derived PAT volumes were comparable to the PAT estimated by CT and that PAT volume was associated with the presence of diabetes independently from other clinical characteristics.

In the study of Kameshima et al. machine learning (ML) methods were used to phenotype heart failure (HF) patients with diastolic dysfunction. These patients from a Japanese HF registry were grouped using two approaches: the first relied on a ML based approach and the second on the grading system recommended by the echocardiographic guidelines for assessing diastolic dysfunction. The ML approach showed to be superior to the echocardiography guideline categories in predicting HF hospitalization in these patients highlighting its value for more accurate risk stratification.

## Coronary Imaging Studies

Novel micro-CT technology was used by Karagiannidis et al. to visualize and analyse extracted thrombus material in 113 patients undergoing primary percutaneous coronary intervention (PCI) for ST-elevation myocardial infarction (STEMI) in a single center. Micro-CT provided non-destructive visualization of aspirated thrombus, allowing reproducible quantification of thrombus volume, surface, and density. The authors found that patients with high angiographic thrombus burden and higher residual thrombus had larger thrombus aspirated; more importantly they showed that, higher thrombus volume and surface area were associated with adverse angiographic outcomes (distal embolization and no-reflow phenomenon).

This analysis highlighted the value of micro-CT in assessing the extracted thrombus burden and its clinical implications. This is especially significant as a meta-analysis of 3 randomized trials demonstrated that there is no prognostic benefit of the routine use of thrombus aspiration in patients with STEMI but in patients with high thrombus burden thrombus aspiration can be beneficial as it is associated with a reduction in cardiovascular mortality and all cause mortality, at the expense of an increased risk of stroke or TIA at 30 days (1).

The review of Liu et al. summarized the methods introduced for coronary plaque segmentation in cardiac CT. Thirty one approaches were presented and categorized into either 2D or 3D plaque extraction methods, with each relevant study within each category subsequently characterized with respect to data quality, methodological innovation and performance.

This detailed review not only highlighted recent advances in CT image but also underscored the need for further research in the field. There is apparently an unmet need to validate the developed methods in large databases and improve plaque extraction accuracy using advanced methods that will allow not only fast and accurate quantification of the atheroma burden but also assessment of plaque vulnerability.

## Myocardial Imaging Studies

Reference ranges are key elements for diagnosis of cardiovascular disease, risk stratification and treatment planning and can be assessed by the distribution of the relevant variable or its predictive value. The study of Leng et al. examined 360 healthy subjects from West China prospectively, and included patients aged 21–79 years with no known cardiovascular disease or uncontrolled cardiovascular risk factors. CMR was used to measure right heart function parameters, including tricuspid annular dynamics as well as longitudinal strain values of the right ventricle and the right atrium. This data allowed for the first time the definition of established age and sex specific reference ranges for tricuspid annular dynamics derived by CMR in healthy individuals which are expected to allow more accurate evaluation of right ventricular function.

Zhao et al. compared LV geometry by three-dimensional (3D) echocardiography and CMR. The authors analyzed 70 subjects (18 patients, 52 healthy volunteers) who had both an 3D-echocardiography and CMR, within 1 h. The authors found there was a systematic bias between the volumes assessed by 3D-echocardiography and those by CMR. They concluded that the systematic signal dropout and the differences in appearances of trabeculae have led to discrepancies in the delineation of LV geometry at anterior and lateral regions resulting in an underestimation of the LV volumes by echocardiography.

### Myocarditis

Suspected acute myocarditis is a leading indication for CMR. State-of-the-art diagnostic criteria for myocarditis are based on the consensus Lake Louise Criteria (LLC) published in 2018 which are an update of the LLC presented in 2009 (2, 3). Li et al. prospectively assessed in 73 patients with a clinically suspected myocarditis which CMR parameters from the 2009 and 2018 LLC can diagnose myocarditis most accurately at a 3.0 Tesla scanner. The gold standard in that study was the presence of inflammation on endomyocardial biopsy (EMB). T1 and T2 mapping techniques showed the highest positive predictive value and were superior compared to the LLC 2018 and LLC 2009 criteria: the area under the curve for T1 mapping + T2 mapping was 0.95 while it was 0.91 for LLC 2018 alone, 0.76 for LLC 2009.

### Right Ventricular Function

Meng et al., examined the value of right ventricular 3D speckle-tracking strain and ejection fraction in predicting heart failure (HF) related hospitalizations or death in patients with HF with preserved ejection fraction (HFpEF). During a median of 17 months 48% of the included patients (*n* = 93) reached the primary endpoint; in that study impaired 3D-speckle-tracking echocardiography values showed to be strongly associated with the occurrence of the primary endpoint of the study suggesting that these variables may be a useful tool for risk stratification in this population.

Furthermore, Tadic et al. noted that right ventricular LS is not routinely assessed, and summarized the existing methodologies for evaluating right ventricular longitudinal strain (LS) using transthoracic echocardiography and presented the evidence supporting its prognostic value in patients with pulmonary hypertension, heart failure and valvular heart disease. For example, a meta-analysis of 1,169 patients with pulmonary hypertension found that those with an RV free-wall strain >- 22% had a significantly higher risk of all-cause mortality. The authors suggest that routine measurement of right ventricular LS may therefore aid risk stratification, with patients possibly being reclassified into higher risk groups when it is taken into account.

With the growing evidence supporting the value of assessing RV function, the ReVISION method was described in 2017 (4). This uses 3D echocardiography (3DE) to quantify the relative contribution of longitudinal, radial, and anteroposterior shortening in the global RV ejection fraction. Tokodi et al. have recently provided details of the updated ReVISION method and algorithm, particularly for volumetric partitioning of the RV cavity and calculation of longitudinal, circumferential, and area strains using 3DE datasets, and also compared the reproducibility of RV function obtained from 3DE and CMR. This method can be used to further evaluate RV function in different disease states, providing further valuable information when compared with routine echocardiographic evaluation.

### Left Ventricular Hypertrophy

A pilot study of 3 patients undertaken by Loncaric et al. provided novel insight into visualizing myocardial remodeling in left ventricular hypertrophy (LVH). Synchrotron radiation-based X-ray phase-contrast imaging (X-PCI) is a research methodology that enables direct measurement of myocardial structure based on changes in X-ray intensity and phase, and enables visualization of individual myocytes and analysis of cardiac microstructure. The authors used 3D X-PCI on myocardial tissue samples obtained during surgical myectomy for three patients with obstructive LVH to assess myocyte organization, 3D connective tissue distribution, and vasculature and compared these findings with the non-invasive cardiac imaging findings (transthoracic echocardiography and CMRI) performed prior to myectomy. The authors found that the myocyte and connective tissue microstructural organization was different in different myocardial pathologies. This is a small pilot study which highlights the potential utility of 3D X-PCI and is expected to provide the substrate for the conduction of larger scale studies that will provide compelling evidence which will justify its routine use in clinical setting (e.g. more compact synchrotrons), with the aim of establishing the etiology of LVH when non-invasive imaging is non-diagnostic.

## Risk Prediction and Optimal Treatment Planning

Rank et al. examined the long term implications of aortic valve replacement on reverse ventricular remodeling. The authors analyzed CMR data acquired 1, 5, and 10 years after aortic valve replacement (AVR) for both aortic stenosis (AS) and aortic regurgitation (AR). The authors found that patients with AR had more long term alteration of myocardial function compared with patients with AS, for example patients with AR had no improvement in myocardial longitudinal strain following AVR, whereas patients with AS show an improvement 1 year after AVR and this remains constant after. The authors conclude that CMR is a useful tool to monitor patients with aortic valve disease, helping to plan AVR at an appropriate stage prior to irreversible structural heart damage.

Moreover, Sahiti et al. use myocardial work analysis derived from echocardiographic and blood pressure measurements, to show that increased LV volume and mass reduce global work efficiency as the cardiac cycle demands more energy. They also found altered myocardial work patterns in hypertensive patients even with normal LV volume and mass. These findings indicate that increased wasted energy combined with LV hypertrophy might be an early sign of hypertensive heart disease; however, further longitudinal studies and more compelling evidence are required to prove this hypothesis.

Gehrsitz et al. described how preoperative planning in pediatric patients with congenital heart disease can be optimized by using cinematic rendering to create three-dimensional (3D) images from two-dimensional (2D) CT or CMR. These 3D images were shown to the surgeon preoperatively on a 2D screen, as a hologram with special glasses (HoloLens®) and as a 3D print. This value of this information was assessed using questionnaires. It was shown that the combination of an extremely photo-realistic presentation via cinematic rendering and the spatial presentation in 3D space via mixed-reality technology enabled detailed assessment of the anatomy and pathology prior to the surgery which was helpful for treatment planning.

The implications of COVID-19 infection on cardiovascular pathology is of great interest. Li et al. demonstrated that routine non-contrast chest CT imaging in patients with COVID-19, performed to evaluate lung disease, also provided useful prognostic implications and prediction of cardiovascular complications. In that study 241 consecutive hospitalized patients with confirmed COVID-19 were enrolled; three cardiac measurements were taken on CT imaging: left atrial diameter (LAD), left ventricular (LV) length and the cardiothoracic ratio (CTR). The authors found that a combination of LAD+LV (cutoff of 11.8 cm) and the CTR (cutoff of 0.8) was a predictor of major adverse cardiovascular events with a sensitivity of 82.6% and specificity of 80.2%.

Tanacli et al. evaluated the value of endomyocardial biopsies (EMB) in COVID-19 compared to CMR to assess myocardial injury. They included 32 patients with persistent symptoms after COVID-19, 22 patients with acute myocarditis not related to COVID-19 and 16 healthy volunteers. These subjects had a baseline CMR as well as a follow-up scan; 10 of the post-COVID-19 and 15 of the non-COVID-19 patients had an EMB. All COVID-19 patients showed impaired myocardial deformation as well as altered functional and anatomical characteristics (e.g., RV mass and T1 times), nevertheless the LLC criteria for myocarditis were only fulfilled in 9% of patients. In these patients traces of previous myocardial inflammation were identifiable without evidence of ongoing inflammation in EMB.

## Conclusion

These papers were the most frequently downloaded and distinguishing manuscripts of 2021. It is clear from the breadth of research and data presented in this topic, that cardiovascular imaging research is a fast moving field, with advances that have potential clinical applications across a wide range of cardiac pathologies, contributing in improved diagnosis, risk stratification and better treatment planning.

## Author Contributions

DH and KC drafted and revised the manuscript. SK and CB initiated the manuscript and guided with editorial comments after review. All authors contributed to the article and approved the submitted version.

## Funding

DH received a CMR specific research grant from the German Centre for Cardiovascular Research (DZHK, Grant Number: 81X3100214).

## Conflict of Interest

The authors declare that the research was conducted in the absence of any commercial or financial relationships that could be construed as a potential conflict of interest.

## Publisher's Note

All claims expressed in this article are solely those of the authors and do not necessarily represent those of their affiliated organizations, or those of the publisher, the editors and the reviewers. Any product that may be evaluated in this article, or claim that may be made by its manufacturer, is not guaranteed or endorsed by the publisher.
